# Validation of Placebo in a Manual Therapy Randomized Controlled Trial

**DOI:** 10.1038/srep11774

**Published:** 2015-07-06

**Authors:** Aleksander Chaibi, Jūratė Šaltytė Benth, Michael Bjørn Russell

**Affiliations:** 1 Head and Neck Research Group, Research Centre, Akershus University Hospital, 1478 Lørenskog, Oslo, Norway; 2 Institute of Clinical Medicine, Akershus University Hospital, University of Oslo, 1474 Nordbyhagen, Oslo, Norway; 3 HØKH, Research Centre, Akershus University Hospital, 1478 Lørenskog, Oslo, Norway

## Abstract

At present, no consensus exists among clinical and academic experts regarding an appropriate placebo for randomized controlled trials (RCTs) of spinal manipulative therapy (SMT). Therefore, we investigated whether it was possible to conduct a chiropractic manual-therapy RCT with placebo. Seventy migraineurs were randomized to a single-blinded placebo-controlled clinical trial that consisted of 12 treatment sessions over 3 months. The participants were randomized to chiropractic SMT or placebo (sham manipulation). After each session, the participants were surveyed on whether they thought they had undergone active treatment (“yes” or “no”) and how strongly they believed that active treatment was received (numeric rating scale 0–10). The outcome measures included the rate of successful blinding and the certitude of the participants’ beliefs in both treatment groups. At each treatment session, more than 80% of the participants believed that they had undergone active treatment, regardless of group allocation. The odds ratio for believing that active treatment was received was >10 for all treatment sessions in both groups (all p < 0.001). The blinding was maintained throughout the RCT. Our results strongly demonstrate that it is possible to conduct a single-blinded manual-therapy RCT with placebo and to maintain the blinding throughout 12 treatment sessions given over 3 months.

Randomized controlled trials (RCTs) are regarded as the gold standard in clinical research. Most pharmacological RCTs are double-blinded and include placebos. However, it is impossible to conduct a double-blinded manual-therapy RCT because the person who applies the intervention is clearly un-blinded. Thus, the question that arises, is whether it is possible to include a placebo arm in a manual-therapy RCT? Previous manual-therapy RCTs on headache either did not include a placebo group or defined placebo as no treatment^1^,^2^,^3^. Two studies evaluated placebo (sham manipulation) in a single treatment session^4^,^5^. Both studies should be commendable for their attempts to blind subjects in the control group; however, they may also be criticized for not applying a true placebo given that a light touch in the affected cervical area may elicit afferent stimulation and application of both active and placebo treatments to the same individual is not ideal^4^,^5^. The participants’ replies were dichotomous “yes” or “no” answers as to whether they believed they received active or placebo treatment, and the results suggested that the participants were blinded.

A few other studies have proposed detuned laser therapy, manipulation instruments and detuned ultrasound as valid sham treatments without proper consideration as to whether participants were adequately blinded^6^,^7^,^8^, and without considering that the placebo intervention should resemble the active intervention^9^. One recent study acknowledged this limitation in their attempt to blind participants in an RCT that included participants with spinal pain^10^. The study group included three modalities during the intervention session: detuned ultrasound, a low-impulse manipulation instrument and the random placement of the investigator’s hand on the participant’s spine to induce manual contact while conducting the ultrasound procedure. However, the study group failed in their attempt to blind the study participants to the group allocation^10^. Thus, significant challenges remain in the development of a valid manual placebo treatment^11^.

Consequently, the question remains as to whether it is possible to provide placebo intervention and maintain the blinding during the course of a typical manual-therapy treatment period, as well as question of how confident the participants are that they have had active treatment.

We investigated chiropractic spinal manipulative treatment (CSMT) versus placebo (sham manipulation) in an RCT involving 12 treatment sessions over 3 months to assess whether it is possible to provide and sustain blinding throughout a full treatment period.

## Material and Methods

### Design

This study was a single-blinded randomized placebo-controlled trial (RCT) involving 12 treatment sessions over 3 months. The study presents an unregistered outcome based on prospective data collection during an otherwise registered study.

### Participants

Participants were recruited from January to September 2013 through the Akershus University Hospital, general practitioners and media advertisement in Akershus and Oslo counties, Norway. Participants received posted information about the project followed by telephone interviews. Eligible participants were between 18 and 70 years of age and had migraine according to the International Classification of Headache Disorders III β (ICHD III β)^12^. Exclusion criteria were contraindications to spinal manipulative therapy (SMT), SMT within the past year, manual therapy from other parties during the treatment period, depression, pregnancy and radiculopathy.

### Randomization

Prepared numbered sealed lots with the two interventions, active and placebo intervention were subdivided into four subgroups by age and gender, i.e., 18–39 and 40–70 years of age and men and women, respectively. The participants drew one lot that allocated them to either the active or the placebo treatment. The blocked randomization procedure minimizes the risk of selection bias and was administered exclusively by an external party without the involvement of the clinical investigator (AC).

### Intervention

Active treatment consisted of CSMT using the Gonstead method, i.e., a specific contact, high-velocity, low-amplitude, short-lever spinal with no post-adjustment recoil that was directed to spinal biomechanical dysfunction (full spine approach) as diagnosed by standard chiropractic tests^13^.

The placebo intervention consisted of sham manipulation, i.e., a broad non-specific contact, low-velocity, low-amplitude sham push manoeuvre in a non-intentional and non-therapeutic directional line. All the non-therapeutic contacts were performed outside the spinal column with adequate joint slack and without soft tissue pre-tension so that no joint cavitations occurred. In some sessions, the participants lay either prone on a Zenith 2010 HYLO bench with the investigator standing at the participant’s right side with his left palm placed on the participant’s right lateral scapular edge with the other hand reinforcing. In other sessions, the investigator stood at the participant’s left side and placed his right palm over the participant’s left scapular edge with the left hand reinforcing, delivering a non-intentional lateral push manoeuvre. Alternatively, the participant lay in the same-side posture as with the active treatment group with the bottom leg straight and the top leg flexed with the ankle resting on the bottom leg’s knee fold, in preparation for a side posture push move, which was delivered as a non-intentional push in the gluteal region. The sham manipulation alternatives were equally interchanged among the placebo participants according to protocol during the 12-week treatment period to strengthen the study validity (Table 1). Both the active and the placebo groups underwent the same structural and motion assessments prior to and after each intervention. No other intervention or advice was given to participants during the trial period. Fifteen minutes was allocated per consultation for each participant. This placebo procedure, innovated by AC, has to our knowledge not previously been used and was not pretested. All participants received intervention by a single experienced chiropractor (AC).

### Blinding

After each treatment session, the participants completed the de-blinding questionnaire administered exclusively by a blinded external trained independent party with no involvement from the clinical investigator, i.e., providing a dichotomous “yes” or “no” answer as to whether active treatment was received. This response was followed by a second question regarding how certain they were that active treatment was received on a 0–10 numeric rating scale (NRS), where 0 represented absolutely uncertain and 10 represented absolutely certainty^14^,^15^.

### Outcome measures

The outcome measures included the rate of successful blinding and the certainty in the participants’ beliefs in both treatment groups.

### Statistical analysis

The dichotomous “yes” and “no” data were presented as percentages with 95% confidence intervals (CI), whereas the continuous 0–10 NRS outcome were presented as the means with 95% CI for each treatment group, i.e., CSMT and placebo.

Time trends in both outcomes were assessed by regression models for repeated measurements, correctly accounting for intra-individual correlations. The dichotomous outcome was analysed by a logistic regression model using the SAS GLIMMIX procedure. Fixed effects for the treatment group and the treatment number were specified. An interaction between the two fixed effects was included into the model to quantify possible differences in trend in the placebo and active treatment groups. Random intercept encountering for within-subject variability was also included in the model.

A linear regression model was fitted for continuous outcome using SAS MIXED procedure. The same fixed effects as in the model above were included into the linear regression model. Additionally, the analyses were stratified by believers vs. non-believers, i.e., participants who believed active treatment was received vs. those not believing that active treatment was received independent of group allocation, by including a fixed effect for dummy variable, which identified the subgroups and the interaction between the dummy and the treatment group as well as the interaction between the dummy and treatment number.

The estimated regression parameters for fixed effects from both models were tabulated together with the standard errors (SEs) and covariances between the parameter estimates. As these models contained interactions, calculation of the odds ratios (OR) and the estimated mean NRS scores with the corresponding 95% confidence intervals (CIs) was somewhat complicated. Therefore, the OR and the estimated mean NRS scores with 95% CIs for each treatment session were calculated and presented graphically.

All statistical analyses were performed by a blinded statistician (JSB) using SPSS v20 and SAS v9.3. P-values below 0.05 were considered statistically significant.

### Ethics

The good clinical practice guidelines were followed^16^. Oral and written information about the project was provided in advance of inclusion and group allocation, i.e., active or placebo treatment, including benefits and possible adverse events (primarily local tenderness and tiredness on the treatment day). Written consent was obtained from all participants. Insurance was provided through the Norwegian System of Compensation to Patients (NPE), an independent national body that compensates patients injured by treatments provided by the Norwegian health service. A stopping rule was defined for withdrawing participants from this study in accordance with the recommendations in the CONSORT extension for Better Reporting of Harms^17^. All adverse events were monitored. Severe adverse event would result in withdrawal from the study and referral to the General Practitioner or hospital emergency department depending on the severity of the event. The investigator was available via the study’s mobile phone at any time throughout study treatment period. The Norwegian Regional Committee for Medical Research Ethics and the Norwegian Social Science Data Services approved the project. All methods were carried out in accordance with the approved guidelines and regulations. The study was registered 2 December 2012 at ClinicalTrials.gov (ID no. NCT01741714).

## Results

Our single-blinded randomized placebo-controlled trial included 70 participants, 35 (6 men and 29 women) in each group. The baseline characteristics were similar in the active and the placebo groups (Table 2).

In total, 772 treatment sessions were completed (390 and 382 in the active and the placebo group respectively, and 68 (8.1%) treatment sessions were missed (30 and 38 in the active and placebo respectively) by 12 subjects in the active group (range 1–10 sessions) and by 10 subjects in the placebo group (range 1–8 sessions), i.e., 2, 0, 3, 5, 6, 5, 3, 5, 7, 8, 10 and 14 missed sessions at each of the 12 consecutive treatment sessions.

Five participants receiving active treatment believed it was placebo at least once during the twelve treatment sessions (1/12, 1/12, 6/11, 8/12, 12/12). Similarly, 11 participants receiving placebo treatment believed it was placebo at least once during the twelve treatment session (1/12, 1/12, 2/12, 2/12, 4/12, 2/4, 6/12, 7/12, 8/12, 9/12, 10/12). These numbers correspond to the number of times a given participant believed they received placebo during the twelve treatment sessions.

**Figure 1:**
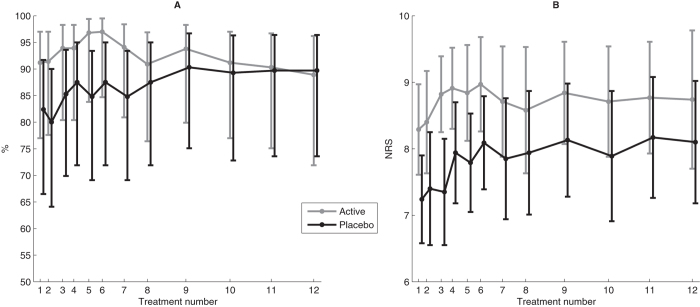
(**A**) The percentages with 95% confidence intervals (CI) of participants believing they had active treatment at each treatment session. (**B**) Mean numeric rating scale (NRS) score with 95% CI for how certain participants were that they received active treatment on a NRS (0–10).

At each treatment session, more than 80% of participants believed they had undergone active treatment regardless of whether they received active or placebo treatment throughout the RCT (Fig. 1A). There was no statistically significant difference between those who had and who had not received SMT previously (p = 0.149). Similarly, there were no statistically significant differences between the treatment groups with respect to previous SMT (p = 0.588), and this result was consistently observed throughout the RCT (Fig. 1B).

**Figure 2:**
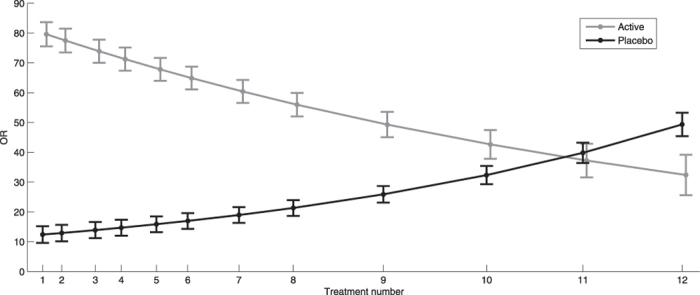
The odd ratios (OR) with 95% confidence intervals (CI) for believing active treatment was received for each consecutive treatment session.

**Figure 3:**
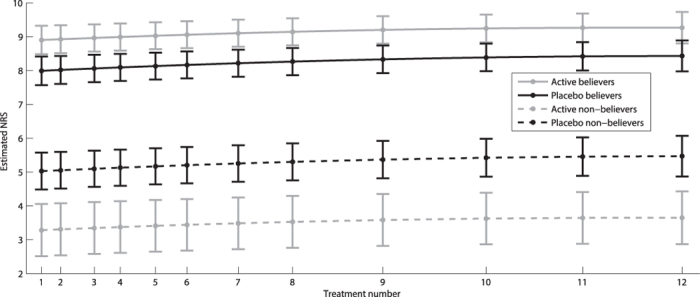
Estimated mean numeric rating score (NRS) (0–10) with 95% confidence intervals (CI) for how certain participants were on receiving active treatment.

Both dichotomous and continuous data showed a strong cluster effect, with correlation over time of 0.6 and 0.7, respectively, justifying the use of regression models for repeated measurements. The odds for believing active treatment was received at baseline were approximately 10 times higher than the odds for not believing placebo was received (p < 0.001). In the active treatment group, these same odds were 73 times higher (p < 0.001). The odds continued to increase in the placebo group for each treatment session, whereas the opposite effect was observed in the active treatment group (Table 3 and Fig. 2). The believers were significantly more certain about their belief than non-believers (Table 4 and Fig. 3). Similarly, the belief among believers and non-believers receiving active treatment was significantly stronger than among those receiving placebo for both comparisons (p < 0.001).

## Discussion

Our main finding is that it is possible to apply placebo and to maintain blinding in a chiropractic manual-therapy single-blinded placebo RCT. The validity of the placebo and the blinding continued throughout the 12 treatment sessions over 3 months. The importance of this finding is emphasized by the fact that all previous manual-therapy studies on headache, whether by RCT or not, lack placebo arms.

At the time of trial registration, we had different thoughts about how to monitor the blinding, and we were uncertain about the best design for this study, as there is no current consensus on the blinding of manual-therapy trials. Video recordings and investigator questioning were considered; however, both methods were rejected, primarily owing to possible biases in relation to the interpretation of the videos and to avoid bias induced by the investigator. Thus, it was finally considered that a brief questionnaire administered by a technical aid after each treatment, would be less biased. The questionnaire was administered after each treatment session because the perception of blinding could change during the course of the investigation. The questions about whether the participant had received “active” or “placebo” intervention along with how strongly they believed that the active treatment was received, were constructed just prior to the baseline period, before the beginning of the intervention.

Two previous manual-therapy RCTs that included participants with headache applied placebo in a single treatment session^4^,^5^. Children naive to SMT received a high-velocity low-amplitude (HVLA) manipulation by a general practitioner without rotation or they received a light touch of the affected specific spinal segments (placebo)^4^. Approximately 20% of the children in either group were unable to tell whether they had received active treatment or placebo, with the remainder guessing the correct treatment, 50% of the times in both groups. Thus, blinding was ascertained; nevertheless, the light touch could have elicited an effect and therefore cannot be regarded as a true placebo. The second experimental study applied active treatment followed by placebo, and placebo followed by placebo intervention^5^. Both treatments were given in a single treatment session. The active treatment was applied on the side of the lesion followed by placebo applied on the other side, i.e., a touch near the target region with positioning of the head and neck, movement and sound timed with treatment delivery that mimicked the active treatment. Correct treatment was anticipated by approximately 50% of participants in each of the two groups. Thus, blinding was ascertained; however, the participants with mechanical neck pain with local tender spots may have received an afferent effect by the light touch directed to the affected area. Thus, the light touch might not constitute a true placebo, as placebo is usually conceived as an inert treatment. We believe that applying both active and placebo treatments during the same treatment session is far from ideal in an RCT, as such a design does not provide meaning in a pharmacological RCT. Moreover, if participants were to receive both treatments during a pharmacological RCT, each treatment would be given separately at different time periods, i.e., a cross-over RCT. One disadvantage of cross-over RCTs is the carryover effect that may also play a role in manual-therapy studies^5^.

Although most manual-therapy RCTs are pragmatic, a few manual-therapy studies have included a placebo intervention^11^, e.g., for mechanical neck pain^18^,^19^, low back pain^20^,^21^,^22^,^23^,^24^, and primary dysmenorrhoea^25^,^26^. However, all these studies omitted from validating blinding. Thus, whether the placebo group was concealed remains unknown.

We observed that the majority of participants believed they had undergone active treatment regardless of whether they received active or placebo intervention, and the response was consistent during the 12 treatment sessions. It has been suggested that only 50% of subjects will believe that they have received active treatment in each group, if the blinding is perfect in a pharmacological double-blinded placebo-controlled RCT^27^. However, this may not hold true in manual-therapy RCTs because the physical stimulus may be more convincing than a tablet^28^,^29^. The fact that we obtained significant success in blinding the participants might be due to the use of interchangeable placebo techniques throughout the treatment period (Table 1). It is generally recommended that the placebo intervention should resemble the active treatment in terms of the procedure, treatment frequency and the time spent with the investigator to allow for similar expectations in both groups^9^. Thus, we believe our success in blinding would have been far less had the patient-provider interaction been skewed.

Furthermore, our full-spine approach resembles the placebo intervention in terms of anatomical locations. Thus, it reduces the risk of disclosing the blinding if participants were to exchange individual experiences. This approach is furthermore substantiated by the large degree of co-occurrence in musculoskeletal disorders. Indeed, it has been postulated that pain in different spinal regions should not be regarded as separate disorders but rather as a single entity^30^.

Our placebo intervention could, however, be criticized because the palpatory procedures and the placebo contacts could have elicited an afferent response. However, This assumption appears unreasonable, particularly considering that there is no single explanation for the placebo effect and because studies contend that several psychological and neurobiological factors contribute to the effect observed^31^,^32^. Furthermore, previous studies have demonstrated that spinal manipulation results in plastic changes in sensorimotor integration within the central nervous system in human participants^33^. Similarly, one recent study reported increased stimulation of afferents with increased duration and amplitude of a spinal manipulation intervention compared with mobilization^34^. Another study found no neurophysiological changes when grade-III mobilization was utilize in asymptomatic participants, i.e., the use of a large-amplitude rhythmic oscillating mobilization technique to the point of limitation in range of movement^35^. While another study have only found reflex surface electromyographic activity to occur after high-velocity low-amplitude SMT as compared to lower-velocity mobilization^36^. Thus, we do not believe that our placebo intervention by itself had any effect other than a placebo effect, particularly considering that all the placebo contacts were made outside the spinal column. Furthermore, initial higher credibility in the active compared to the placebo intervention might carry the risk of a better outcome merely due to a higher positive expectation. However, although the results might present that impression initially, the opposite effect was seen as the trial proceeded. Thus, issues related to expectations of improvement are believed to be minimal.

Previously, no consensus existed among experts, including both clinicians and academics, regarding an appropriate placebo for a clinical trial of SMT^37^. However, this is not a matter of what can be agreed upon but rather what can be scientifically proven to be a valid placebo intervention.

As many manual therapies, e.g., physiotherapy, chiropractic, and osteopathy along with other practices including massage therapy, utilize spinal joint mobilization and manipulation in treating musculoskeletal pain and disability, our placebo procedure, including the brief de-blinding questionnaire, may be easily replicated in future RCTs.

## Conclusion

The results of our chiropractic manual-therapy single-blinded placebo RCT indicate that it is possible to include a valid placebo group, considering that we demonstrated successful blinding during 12 treatment sessions over three months in both active and placebo groups.

## Additional Information

**How to cite this article**: Chaibi, A. *et al.* Validation of Placebo in a Manual Therapy Randomized Controlled Trial. *Sci. Rep.* **5**, 11774; doi: 10.1038/srep11774 (2015).

## Figures and Tables

**Table 1:** Fixed treatment schedule for the placebo group.

Week 1: two sessions	Week 2:two sessions	Week 3: two sessions	Week 4: one session	Week 5: one session
Reinforced left and right scapula push sequentially	Bilaterally scapula push and left gluteal push	Reinforced right and left scapula push sequentially	Reinforced right scapula and left gluteal push	Bilaterally scapula push
**Week 7: one session**	**Week 9: one session**	**Week 11: one session**	**Week 12: one session**	
Bilaterally scapula push and right gluteal push	Bilaterally scapula push	Reinforced left scapula and right gluteal push	Bilaterally scapula and left gluteal push	

**Table 2:** Baseline demographics and characteristics.

	CSMT	Placebo
Number	35	35
Age (range)	41.2 ± 11.3 (19-63)	39.6 ± 9.7 (18-65)
M/F	6/29	6/29
Migraine without aura	33	31
Migraine with aura	9	10
Duration (years with migraine)	22.6 ± 13.6	20.8 ± 10.7
Frequency (30 days/month)	7.6 ± 4.3	7.8 ± 5.0
Co-morbid tension-type headache (%)	25 (71.4%)	27 (77.1%)
Previously received CSMT (%)	12 (34.3%)	13 (37.1%)

*CSMT = chiropractic spinal manipulative therapy; ± = standard deviation.

**Table 3:** Coefficients from a logistic regression model for repeated measurements with standard errors (SE) and covariances between parameters.

	Coefficient (SE)	p-value	
Intercept	2.52 (0.53)	<0.001	
Tr.group	1.86 (0.89)	0.036	
Tr.number	0.02 (0.01)	0.043	
Tr.group by Tr.number	−0.03 (0.01)	0.049	
			
Covariances			
	Tr.group	Tr.number	Tr.group by Tr.number
Intercept	−0.276	−0.001	0.001
Tr.group		0.001	−0.006
Tr.number			−0.00007

*Tr = treatment.

**Table 4:** Coefficients from a linear regression model for repeated measurements with standard errors (SE) and covariances between parameters.

	Coefficient (SE)	p-value				
Intercept	5.03 (0.28)	<0.001				
Tr.group	−1.74 (0.48)	<0.001				
Tr.number	0.01 (0.01)	0.058				
Tr.number by Tr.number	−0.00006 (0.00006)	0.332				
Tr.group by Tr.number	−0.0009 (0.003)	0.749				
Believer	2.96 (0.22)	<0.001				
Tr.group by Believer	2.65 (0.42)	<0.001				
						
Covariances						
	Tr.group	Tr.number	Tr.number by Tr.number	Tr.group by Tr.number	Believer	Tr.group by Believer
Intercept	−0.076	−0.0003	0.000003	0.00008	−0.039	0.040
Tr.group		0.0001	−0.0000008	−0.0002	0.039	−0.158
Tr.number			−0.0000006	-0.000005	−0.00008	−0.000006
Tr.number by Tr.number				0.000000004	0.0000003	0.0000007
Tr.group by Tr.number					0.00005	−0.000009
Believer						−0.047

*Tr = treatment.
